# miR-21 Protects Against Ischemia/Reperfusion-Induced Acute Kidney Injury by Preventing Epithelial Cell Apoptosis and Inhibiting Dendritic Cell Maturation

**DOI:** 10.3389/fphys.2018.00790

**Published:** 2018-06-26

**Authors:** Nana Song, Ting Zhang, XiaLian Xu, Zhihui Lu, Xiaofang Yu, Yi Fang, Jiachang Hu, Ping Jia, Jie Teng, Xiaoqiang Ding

**Affiliations:** ^1^Division of Nephrology, Zhongshan Hospital,Fudan University, Shanghai, China; ^2^Shanghai Medical Center of Kidney, Shanghai, China; ^3^Shanghai Institute of Kidney and Dialysis, Shanghai, China; ^4^Shanghai Key Laboratory of Kidney and Blood Purification, Shanghai, China; ^5^Hemodialysis Quality Control Center of Shanghai, Shanghai, China

**Keywords:** microRNA-21, hypoxia induced factor, dendritic cells, apoptosis, renal injury

## Abstract

Renal tubular injury and innate immune responses induced by hypoxia contribute to acute kidney injury. Accumulating evidence suggests that miR-21 overexpression protects against kidney ischemia injury. Additionally, miR-21 emerges as a key inhibitor in dendritic cell maturation. Thus, we hypothesized that miR-21 protects the kidney from IR injury by suppressing epithelial cell damage and inflammatory reaction. In this study, we investigated effects of miR-21 and its signaling pathways (PTEN/AKT/mTOR/HIF, PDCD4/NFκ-B) on kidney ischemia/reperfusion (IR) injury *in vitro* and *in vivo*. The results revealed that IR increased miR-21, HIF1α, and 2α expression *in vivo* and *in vitro*. MiR-21 interacted with HIF1α and 2α through the PTEN/AKT/mTOR pathway. Moreover, inhibition of miR-21 activated PDCD4/NFκ-B pathways, which are critical for dendritic cell maturation. Renal IR triggers local inflammation by inducing the dendritic cell maturation and promoting the secretion of IL-12, IL-6, and TNF-α cytokines. Knockdown of miR-21 intensified the effect of IR on tubular epithelial cell apoptosis and dendritic cell maturation. Our results suggested that IR-inducible miR-21 protects epithelial cells from IR injury via a feedback interaction with HIF (PTEN/AKT/mTOR/HIF/miR-21) and by inhibiting maturation of DCs through the PDCD4/NF-κB pathway. These findings highlight new therapeutic opportunities in AKI.

## Introduction

Acute kidney injury (AKI) is a severe and even fatal disease and with high incidence each year. Early AKI diagnosis and therapy are critical for recovery. Ischemia/reperfusion injury (IRI) represents the primary pathogenesis of AKI, leading to tubular epithelial cell necrosis and innate immune responses. Despite enormous efforts to assess the mechanisms of AKI, early diagnosis is difficult to achieve, and therapeutic choices remain limited.

MicroRNA (miR) is a large family of single-stranded, small, noncoding RNAs that regulate expression of their target mRNAs (He and Hannon, 2004). Among this family, miR-21 promotes proliferation, inhibits apoptosis, regulates inflammation and is related to serial renal diseases, such as renal cell carcinoma, diabetic nephropathy, renal fibrosis and acute renal injury (Zhang and Shu, 2016; Ichii and Horino, 2018). Up-regulation of miR-21 is associated with the protective effect of delayed ischemic and xenon preconditioning of the kidney (Xu et al., 2012, 2014; Jia et al., 2015). Dysfunctional miR-21 expression plays a role in the mechanisms responsible for AKI progression (Li et al., 2013). The expression of miR-21 increased after IRI, which is considered a self-protective mechanism (Godwin et al., 2010; Li et al., 2013).

Hypoxia-inducible factors (HIF) play crucial roles in the adaptation of the response to ischemic or hypoxic conditions (Heyman et al., 2015). Accumulating evidence suggested that both HIF1α and HIF2α are protective in renal IR injury (Hill et al., 2008). A putative HIF-1α consensus binding site was identified in the miR-21 promoter region. HIF-1α binding to miR-21 activates miR-21 expression and subsequently up-regulates miR-21-mediated increases in HIF-1α expression in cardiomyocytes (Liu et al., 2014). However, how HIF1α/ HIF2α and miR-21 interact in renal epithelial cells remains unclear.

In addition to hypoxia-induced renal epithelial cell injury, innate immune responses are also critical in the pathogenesis of ischemic acute kidney injury. Dendritic cells (DCs) represent a major population of immune cells in the kidney; these cells are key initiators and effectors of the innate immune system (Tadagavadi and Reeves, 2010). Immature DCs contribute to immune tolerance. However, mature DCs induce immune activation (Yang et al., 2015). Recent research revealed that miR-21 is negatively correlated with the maturation of DCs (Al Akoum et al., 2015). The miR-21/nuclear factor-kappa B (NF-κB) axis plays an important role in immune regulation (Sheedy, 2015). MiR-21 inhibits the programmed cell death 4 (PDCD4)/NF-κB pathway, and NF-κB is a positive regulator for maturation of DCs (Sheedy et al., 2010; Lopez et al., 2015; Su et al., 2015). In this context, miR-21 overexpression in kidney may prevent DC maturation and repress the inflammatory response.

Thus, we hypothesize that up-regulation of miR-21 upon IR conditioning may protect the kidneys from injury by suppressing epithelial cell damage and by inhibiting dendritic cell maturation. In the present study, we investigated the effect of miR-21 and downstream (phosphatase and tensin homolog deleted on chromosome 10 (PTEN)/protein kinase B (AKT)/mammalian target of rapamycin (mTOR)/HIF, PDCD4/NFκ-B) pathways on kidney injury using *in vitro* (hypoxia/reoxygenation, HR) and *in vivo* (ischemia/reperfusion, IR) experimental models. Our present findings confirm this hypothesis and demonstrate that miR-21 up-regulation represents an essential self-protective factor in IRI, revealing new therapeutic opportunities in AKI.

## Materials and methods

### Reagents and materials

Information regarding reagents and materials used in this protocol is provided in the Supplemental Materials (Table 1).

**Table 1:** Reagents and materials.

**Reagents and materials**	**Manufacturers**	**Cat. no**.
Cobalt dichloride (CoCl_2_)	Sigma-Aldrich, Inc. (St. Louis, MO, USA)	60818
L-Mimosine (from Koa haole seeds)	Sigma-Aldrich, Inc. (St. Louis, MO, USA)	M0253
LY294002	Sigma-Aldrich, Inc. (St. Louis, MO, USA)	L9908
Rapamycin	Gene Operation (Shanghai, China)	IPA1021
Locked nucleic acid (LNA)-modified anti-scrambled oligonucleotides	Exiqon (MA, USA)	synthesized
Locked nucleic acid (LNA)-modified anti-miR-21 oligonucleotides	Exiqon (MA, USA)	synthesized
Dulbecco's modified Eagle's medium/nutrient mixture F-12 (DMEM/F12)	KeyGEN BioTECH (Nanjing, China)	KGM12500
AnnexinV-FITC/PI staining kit	KeyGEN BioTECH (Nanjing, China)	KGA105
Fetal bovine serum (FBS)	GIBCO (MA, USA)	0100147
AKT antibodies	Cell Signaling Technology (Danvers, MA)	4691s
phospho-AKT (Ser 473) antibodies	Cell Signaling Technology (Danvers, MA)	4060s
HIF1α antibodies	Cell Signaling Technology (Danvers, MA)	14179
HIF2α antibodies	Abcam (MA, UAS)	ab179825
mTOR antibodies	Abcam (MA, UAS)	ab137133
p-mTOR antibodies	Abcam (MA, UAS)	ab134903
Anti-S6K1 antibody	Abcam (MA, UAS)	ab186753
Phospho-p70 S6 Kinase (Thr389)	Cell Signaling Technology (Danvers, MA)	9234
PTEN antibodies	Abcam (MA, UAS)	ab170941
NF-κB p65 (Ser536) antibodies	Abcam (MA, UAS)	ab28856
NF-κB p65 antibodies	Abcam (MA, UAS)	ab16502
CCR-7 antibodies	Abcam (MA, UAS)	ab32527
U6 TaqMan™ MicroRNA Assays	Thermo Fisher Scientific (MA, USA)	001973
miRNA-21 TaqMan™ MicroRNA Assays	Thermo Fisher Scientific (MA, USA)	000397
Lipofectamine 2000	Thermo Fisher Scientific (MA, USA)	11668030
Opti-MEM serum-free media	Thermo Fisher Scientific (MA, USA)	31985
Trizol	Thermo Fisher Scientific (MA, USA)	15596-026
Human HIF-1α siRNAs	Santa Cruz Biotechnology, Inc. (TX, USA)	sc-44225
Human HIF-2α (EPAS-1) siRNAs	Santa Cruz Biotechnology, Inc. (TX, USA)	sc-35316
Human scramble siRNAs	Santa Cruz Biotechnology, Inc. (TX, USA)	sc-37007
Antibody against GAPDH	Santa Cruz Biotechnology, Inc. (TX, USA)	sc-137179
Human HIF1α primers (Forward: 5'TGCAACATGGAAGGTATTGC3'; Reverse: 5'TTCACAAATCAGCACCAAGC3')	Sangon Biological Engineering Co. Ltd. (Shanghai, China)	synthesized
Human HIF2α primers (Forward: 5' TGGTAGCCCTCTCCAACAAG'; Reverse: 5'TCATCCGTTTCCACATCAAA')	Sangon Biological Engineering Co. Ltd. (Shanghai, China)	synthesized
Human GAPDH primers	Sangon Biological Engineering Co. Ltd. (Shanghai, China)	B661104
PrimeScript reverse transcription reagent Kit	TaKaRa Biotechnology Co., Ltd. (Kyoto, Japan)	RR037A
TaqMan®MicroRNA reverse transcription reagent Kit	Thermo Fisher Scientific (MA, USA)	4366596
SYBR® Premix Ex Taq™	TaKaRa Biotechnology Co., Ltd. (Kyoto, Japan)	RR071A
Creatinine Assay Kit	Bioassay system (CA, UAS)	DICT-500
PE-cy5-labeled CD45 antibodies	eBioscience (CA, USA)	35-0415-80
PE-labeled CD11c antibodies	eBioscience (CA, USA)	12-0114-82
FITC-labeled MHC-2 antibodies	eBioscience (CA, USA)	11-5321-81
APC-labeled CD80 antibodies	eBioscience (CA, USA)	17-0801-81
Mouse IL-12 p70 Quantikine ELISA Kit	R&D Systems, Inc. (MN, USA)	M1270
Mouse IL-6 Quantikine ELISA KIT	R&D Systems, Inc. (MN, USA)	M6000B
Mouse TNF-α ELISA KIT	R&D Systems, Inc. (MN, USA)	MTA00B
Cleaved-Caspase 3 antibody	Cell Signaling Technology (Danvers, MA)	9579

### Animals

Male C57BL/6 mice (16 weeks, weighing 25–30 g) were obtained commercially (Animal Center of Fudan University, Shanghai, China), housed in acrylic cages with shredded corn-cob bedding in an acclimatized room (12/12 h light/dark cycle; 22 ± 3°C) and provided water and mouse breeder chow *ad libitum* according to standard protocols for animal care. The procedures used were approved by the Institutional Animal Care and Use Committee of Fudan University and adhered strictly to the National Institutes of Health Guide for the Care and Use of Laboratory Animals.

### IR model in mouse and pretreatment with reagents

Renal ischemia was performed in 16-week-old male C57BL/6J mice as previously described (Xu et al., 2012). Briefly, mice were anesthetized by intraperitoneal injection of 4% phenobarbitone (10 μl/g body weight). After performing midline laparotomy, bilateral renal pedicles were clamped for 35 min using an atraumatic vascular clamp and then perfused. After this process, the incision was closed. The sham-operated group underwent the same surgical procedure without clamping of the renal pedicle. Rectal temperature was maintained at 37°C. After reperfusion for 24 h, kidneys were harvested.

L-Mimosine and CoCl_2_ were dissolved and diluted in 10% NaHCO_3_ (pH adjusted to 7.4 with HCl) and ddH_2_O, respectively. L-Mimosine (50 mg/kg) or CoCl_2_ (20 mg/kg) were administered intraperitoneally 6 h before IR surgery. The dosages were selected based on a previous study (Kerendi et al., 2006; Fang et al., 2013). Injection of 10% NaHCO_3_ (pH adjusted to 7.4 with HCl) or ddH_2_O served as vehicle control.

LNA-modified anti-miR-21 oligonucleotides were diluted in saline (5 mg/ml) and administered into the tail vein (10 mg/kg) 1 h before kidney IR surgery to knockdown miR-21 expression (Jia et al., 2013). Injection of the same dose of LNA-modified anti-scrambled served as control.

### HR*in vitro*and cell treatments

Human proximal tubular cell lines (HK-2 cells, ATCC) were cultured in DMEM/F12 medium containing 10% FBS in a humidified atmosphere with 5% CO_2_ at 37°C until confluence. For hypoxia/reoxygenation, cells were cultured in a hypoxic atmosphere containing 1% O_2_, 94% N_2_, 5% CO_2_ (Air Liquide) for 6 h and then transferred to normoxic conditions with 21% O_2_ and maintained for 30 min or 1 h. Alternatively, cells were treated with 150 μM cobalt chloride or 500 μM L-mimosine to chemically mimic hypoxic conditions for 6 h. The same volume of vehicle was added as a control.

LY294002 and rapamycin were dissolved in DMSO and diluted with 0.9% NaCl. Cells were pretreated with LY294002 (50 μM) for Akt inhibition or rapamycin (20 nM) for mTOR inhibition for 1 h prior to HR.

### Transient transfection

HK-2 cells were transfected with LNA-modified anti-miR-21 or scramble oligonucleotides at final concentrations of 50 nM using Lipofectamine 2000. The transfection mixture was dissolved in Opti-MEM serum-free media. At the time of transfection, cells were seeded in medium with 10% fetal bovine serum and no antibiotics. Six hours after transfection, the medium was exchanged for full-growth medium (Jiao et al., 2016).

To knockdown HIF-1a and/or HIF-2α expression, the synthesized human HIF-1α and HIF-2α siRNA oligonucleotides were transfected into cells along with the scrambled oligonucleotides using Lipofectamine 2000 according to the manufacturer's protocol. Transfected cells were subjected to HR after 48 h of transfection.

### Western blotting

Western blotting was performed as previously described (Song et al., 2015). Cells and renal tissues were homogenized and centrifuged at 12,000 g for 15 min at 4°C, and the supernatant was collected. Samples were loaded and separated on a sodium dodecyl sulfate-polyacrylamide gel and transferred to a PVDF membrane. The membrane was blocked with 5% nonfat milk and incubated with the primary antibodies against PTEN (1:500), total AKT (1:1,000), phospho-AKT (1:1,000), total mTOR (1:1,000), phospho-mTOR (1:1,000), S6K (1:1,000), phospho-S6K (1:1,000), HIF-1α (1:500), HIF-2α (1:500), PDCD4 (1:500), phospho-NF-κB p65 (1:1,000), NF-κB p65 (1:1,000), CCR-7 (1:1,000) and GAPDH (1:10,000) overnight at 4°C. The membrane was then incubated with HRP-conjugated secondary antibodies and developed using chemiluminescent horseradish peroxidase substrate. The results were normalized to GAPDH.

### Quantitative RT-PCR

Total RNA from harvested cells or renal tissue was extracted using Trizol according to the manufacturer's instruction. Complementary DNA (cDNA) was obtained from reverse transcription of total RNA using the PrimeScript reverse transcription reagent Kit. For miR-21 and U6, the total RNA sample was reverse transcribed into cDNA using U6 and miR-21 TaqMan™ MicroRNA Assays and TaqMan MicroRNA Reverse Transcription Kit following the manufacturer's instructions. The cDNA sample was used as a template for real-time polymerase chain reaction (PCR) (SYBR™ Premix Ex Taq) following the manufacturer's instructions. The primer pairs used are presented in the Reagents and Materials section. GAPDH and U6 were used as endogenous controls for mRNAs and miRNAs, respectively. The relative gene expression was calculated using the ΔΔCt method. Relative mRNA levels were expressed as 2^−ΔΔ*Ct*^ (Tn) and ratios to control (Jia et al., 2015).

### Assessment of serum creatinine

Blood samples were obtained via cardiac puncture. Plasma creatinine was measured using Quantichrom Creatinine Assay Kit employing the improved Jaffe method.

### Histopathological examinations and immunohistochemical staining

Kidney slices were fixed in 10% formalin, embedded in paraffin, cut into 5-μm sections, and stained with Periodic Acid Schiff (PAS) Stain Kit or cleaved-caspase 3 immunohistochemical staining. For PAS staining, histologic injury scores were evaluated under light microscopy by a pathologist blinded to the origin of preparations and determined using a scoring system, as described in the previous study (Dai et al., 2016). Injury was scored according to the percentage of damaged tubules as follows: no injury (0), mild: less than 25% (1), moderate: less than 50% (2), severe: less than 75% (3), and very severe: greater than 75% (4). Immunohistochemical staining was performed as described previously (Song et al., 2012, 2016). After incubation with first antibody against claved-caspase3 (1:100), the reaction was detected with an avidin-biotin-HRP complex (ABC) immunodetection kit and examined with light microscopy. Relative optical density (ROD) [(positive staining-background)/background] of positive-stained cells were calculated in six representative sections from each animal in a blinded manner to the treatment using Image Measure Version 1.0 software (Fudan University, Shanghai, China).

### Preparation of single-cell suspensions from kidney

Single cell suspensions of kidney were isolated as described previously (Tadagavadi and Reeves, 2010). Briefly, renal tissue was minced into smallpieces followed by digestion and disruption in DMEM mixture containing 2 mg/ml collagenase type I for 45 min at 37°C to obtain a single-cell suspension. The suspension was passed through a 200-mesh (74 μm) filter into a 15-ml tube and was centrifuged at 1,200 rpm (300 g) for 10 min. The resulting pellet was incubated in red blood cell lysis buffer to remove red blood cells.

### Flow cytometry

The AnnexinV-FITC/PI staining kit was used to assess HK-2 cell apoptosis according to the manufacturer's instructions. To measure the abundance and maturity of dendritic cells in kidney, single cell suspensions from kidney were stained using the following fluorochrome-labeled antibodies: anti-CD45 (PE-CY5), CD11c (PE), MHC-2 (FITC), and CD80 (APC). Flow cytometry was performed using a FACSCalibur (Becton Dickinson, Heidelberg, Germany) and analyzed using FlowJo 10.0 software.

### Enzyme-linked immunosorbent assay

The kidney tissues were homogenized in PBS and centrifuged to obtain supernatants. The concentration of protein in the supernatants was measured by a BCA protein assay kit and adjusted to 2 × 10^4^ μg/ml by PBS. IL-12, IL-6, and TNF-α levels were detected by commercially available enzyme-linked immunosorbent assay (ELISA) kit (R&D Systems, Minneapolis, MN) following the manufacturer's protocol.

### Statistics

Statistical analysis was performed using Statistical Package for the Social Sciences (SPSS) version 16.0. Data are presented as the mean ± SE. For comparison between two groups, two-tailed, unpaired *t*-tests were used. For multiple comparisons, one-way ANOVA was applied followed by *post hoc* Student's and Newman-Keuls tests where appropriate. The scores were presented as a class variable and analyzed using the Kruskal-Wallis nonparametric test. All comparisons were two-tailed, and *P* < 0.05 was considered significant.

## Results

### The protective role of mIR-21 on IRI

**Figure 1:**
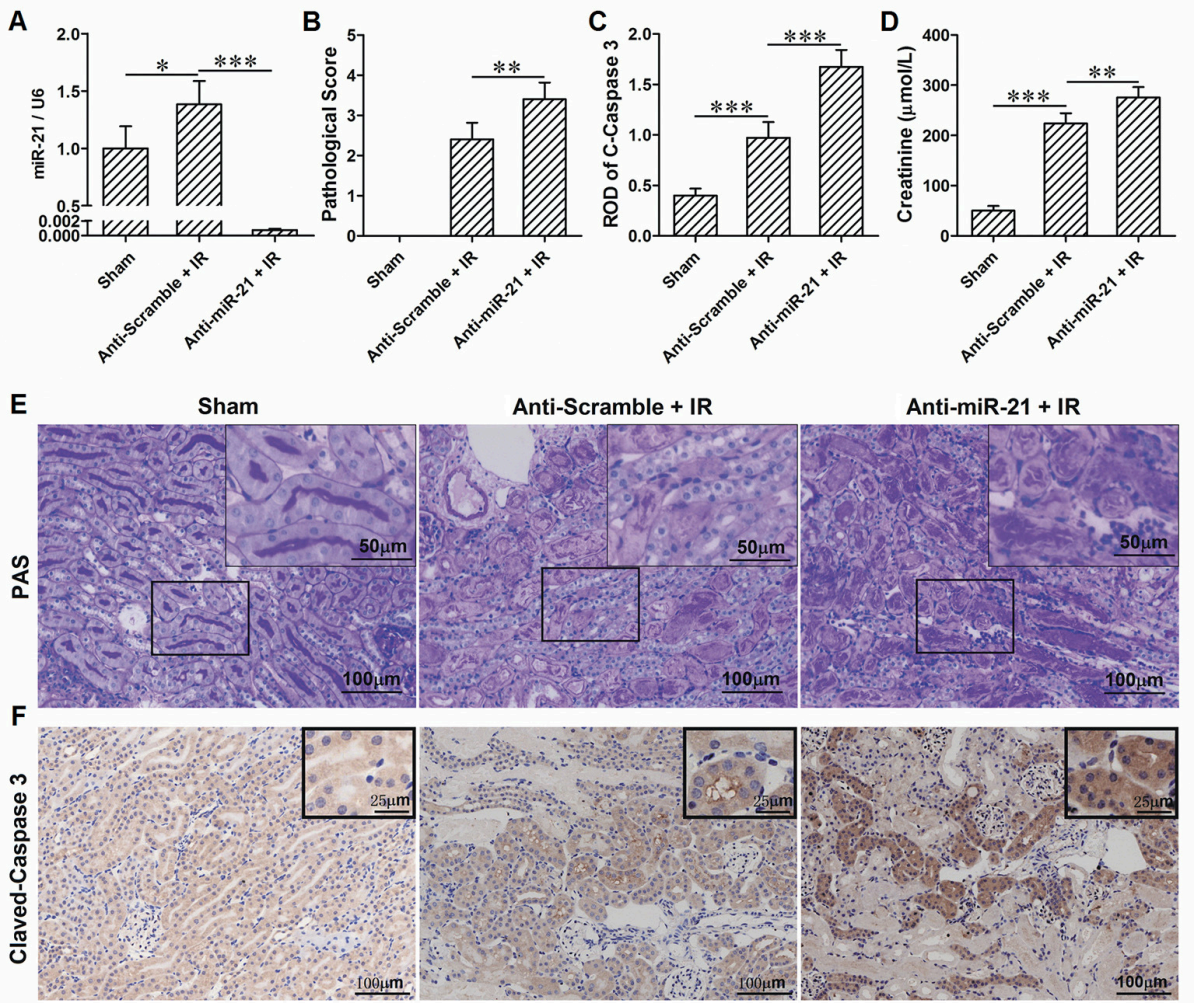
IR induces up-regulation of miR-21 in kidney and knockdown of miR-21 aggravates renal IRI. **(A)** Expression level of miR-21 in the kidney was higher in the IR group compared with sham group. Injection of LNA-modified anti-miR-21 (10 mg/kg) through the tail vein 1 h before kidney IR surgery significantly blocked expression of miR-21. **(B)** PAS staining was performed to access the injury of the kidney. IR-induced kidney damage is more evident in the miR-21 interfered animals. **(C)** immunohistochemical analysis of Cleaved-Caspase 3 was performed to access the apoptosis of the tubular epithelia. Anti-miR-21 exacerbated IR-caused epithelia apoptosis. **(D)** Level of creatinine in the serum was measured to evaluate renal function. Anti-miR-21 aggravated the decline of renal function. **(E)** Typical visual field of PAS staining. **(F)** Typical visual field of immunohistochemical staining of Cleaved-Caspase 3. *P < 0.05, **P < 0.01, *** < 0.001, *n* = 6.

**Figure 2:**
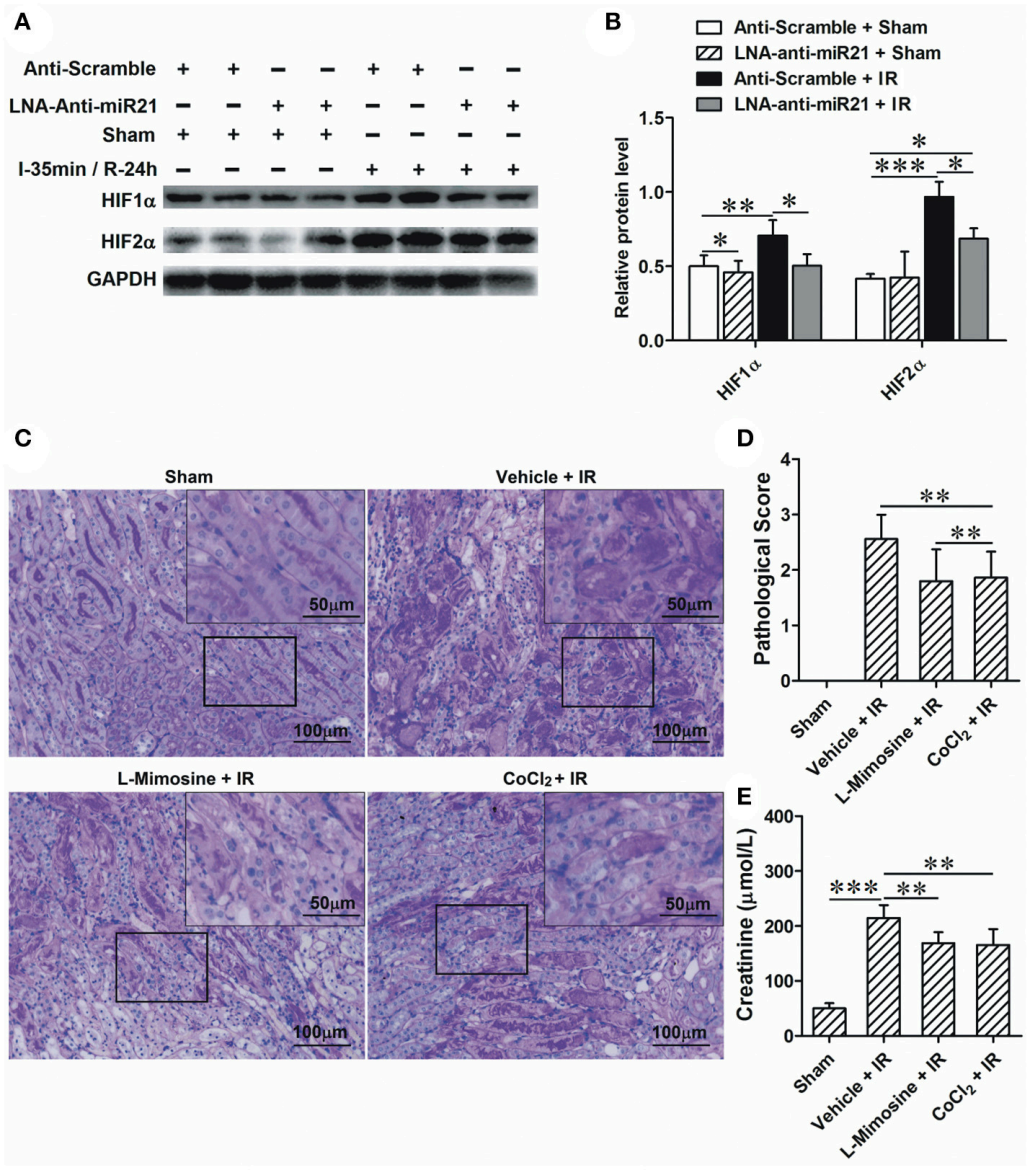
miR-21 regulates IRI by modulating HIF-1α and HIF-2α expression. **(A)** Injection of LNA-modified anti-miR-21 (10 mg/kg) through the tail vein 1 h before kidney IR surgery or sham operation. Blocking miR-21 attenuated IR-induced up-regulation of HIF-1α and HIF-2α in the kidney. **(B)** Group data from **A**. **(C)** L-Mimosine (50 mg/kg) or CoCl_2_ (20 mg/kg) was injected intraperitoneally 1 h before kidney IR surgery. PAS staining was performed to access the injury of the kidney. IR-induced kidney damage is mainly presented as epithelial denudation and dilation. The injury is lower in the L-Mimosine or CoCl_2_-treated animals. **(D)** group data from **C**. **(E)** Level of creatinine in the serum. *P < 0.05, **P < 0.01, *** < 0.001, *n* = 6.

To investigate the roles of miR-21 in response to IRI, we analyzed miR-21 expression under ischemia/reperfusion conditions *in vivo*. We found that IR induced miR-21 expression (Figure 1A). The kidney injury was assessed by PAS staining, cleaved-caspase 3 staining and creatinine serum levels. As the results indicate, renal function is exacerbated in animals subject to LNA-anti miR-21 interference compared with scramble control (Figures 1B–F). In addition, we investigated the effect of miR-21 on HIF-1α and HIF-2α expression after IRI. IR induced HIF-1a and HIF-2α expression in the kidney, and knockdown of miR-21 by LNA-anti-miR-21 reduced the expression of both HIF-1α and HIF-2α in IR-challenged kidney (Figures 2A,B). To confirm the vital role of HIF, L-mimosine and CoCl_2_ were used to inhibit hydrolysis of HIF, and kidney injury was analyzed based on PAS staining and serum creatinine levels. L-Mimosine or CoCl_2_ alleviated kidney damage induced by IR (Figures 2C–E).

### MiR-21 regulated the renal inflammatory response by mediating maturation of renal dendritic cells

**Figure 3:**
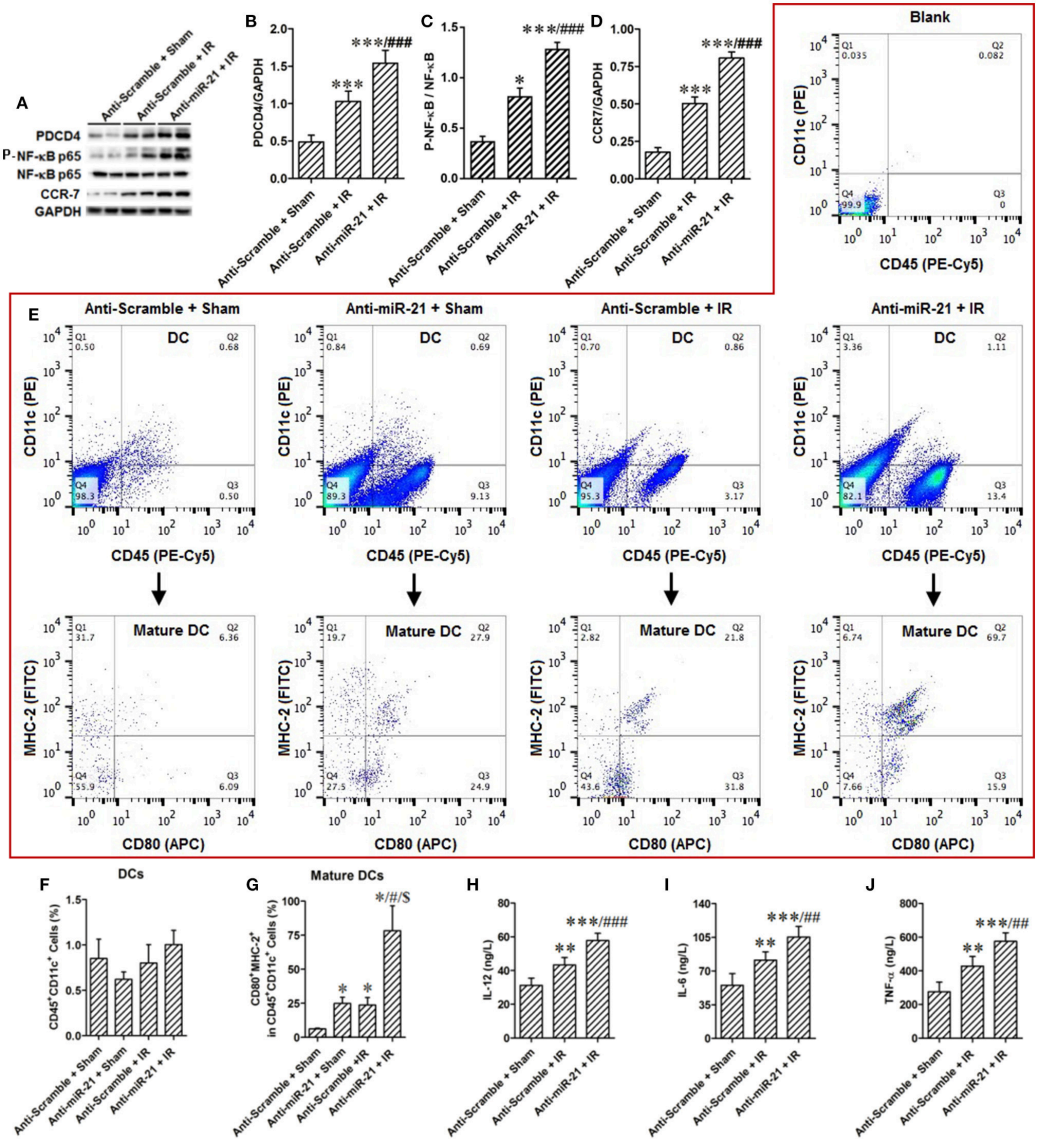
Knockdown of miR-21 aggravate renal inflammatory response by promoting maturation of renal dendritic cells. **(A–D)** MiR-21 was inhibited by injection of LNA-modified anti-miR-21 before kidney IR surgery or sham operation. The effect of miR-21 on activation of PDCD4/NF-κB/CCR-7 pathway that was essential in maturation of DCs was assessed in the IR treated kidney. IR activated PDCD4/NF-κB/CCR-7 pathway, and knockdown of miR-21 further elevated IR-inducible PDCD4/NF-κB/CCR-7. **P < 0.01, *** < 0.001, compared with Anti-scramble + Sham; ^##^*P* < 0.01, ^###^*P* < 0.001, compared with Anti-scramble + IR, *n* = 6. **(E)** The percentage of DCs or mature DCs in the single cell suspensions of the kidney was measured by flow cytometry. Either knockdown of miR-21 or IR promoted maturation of DCs, but not alter the filtration of total DCs. Moreover, knockdown of miR-21 further aggravated IR-induced maturation of DCs. **(F,G)** Group data from **(E)**. *P < 0.05, compared with Anti-scramble + Sham; ^#^*P* < 0.05, compared with Anti-miR-21 + Sham; ^$^*P* < 0.05, compared with Anti-scramble + IR, *n* = 3. **(H–J)** The produce of proinflammatory factors IL-12, IL-6, TNF-α which were secreted from mature DCs was assessed by ELISA. As expected, IR increased and knockdown of miR-21 further raised their content. **P < 0.01, *** < 0.001, compared with Anti-scramble + Sham; ^##^*P* < 0.01, ^###^*P* < 0.001, compared with Anti-scramble + IR, *n* = 6.

Dendritic cells (DCs) are the most abundant resident leukocytes present in the kidney. To explore the mechanism of preventing the effect of miR-21 on renal dendritic cell maturation, we examined the products of PDCD4 and NF-κB and DC maturation in the kidney. IR increased PDCD4 and NF-κB levels; anti-miR21 further increased these levels (Figures 3A–C). To determine the effect of miR-21 on DC maturation under conditions of renal IR injury, we examined expression of CCR-7 (marker of mature DCs) in the kidney as well as the percentage of DCs (CD45^+^/*CD*11*c*^+^) and mature DCs (CD45^+^/*CD*11*c*^+^/*CD*80^+^/*MHC*−2^+^) among single-cell suspensions isolated from kidney. IR increased CCR-7 levels, and anti-miR21 further increased this level (Figure 3D). IR did not increase infiltration of DCs but induced mature DCs in the kidney. Additionally, anti-miR-21 enlarged the effect of IR in the maturation of DCs. Moreover, anti-miR-21 in animals without IR surgery also promotes maturation of kidney DCs (Figures 3E–G). These results indicated that inhibiting miR-21 improves kidney DC maturation via the PDCD4/NF-κB pathway. To confirm the effect of miR-21 on IR-induced inflammatory response, we measured pro-inflammation cytokines, such as IL-6, IL-12, and TNFα, which are secreted by mature DCs in the kidney. As expected, anti-miR-21 increased the expression of these cytokines (Figure 3H–J).

### The feedback loop between miR-21 and HIFs is involved in hr-induced renal epithelial cell injury

**Figure 4:**
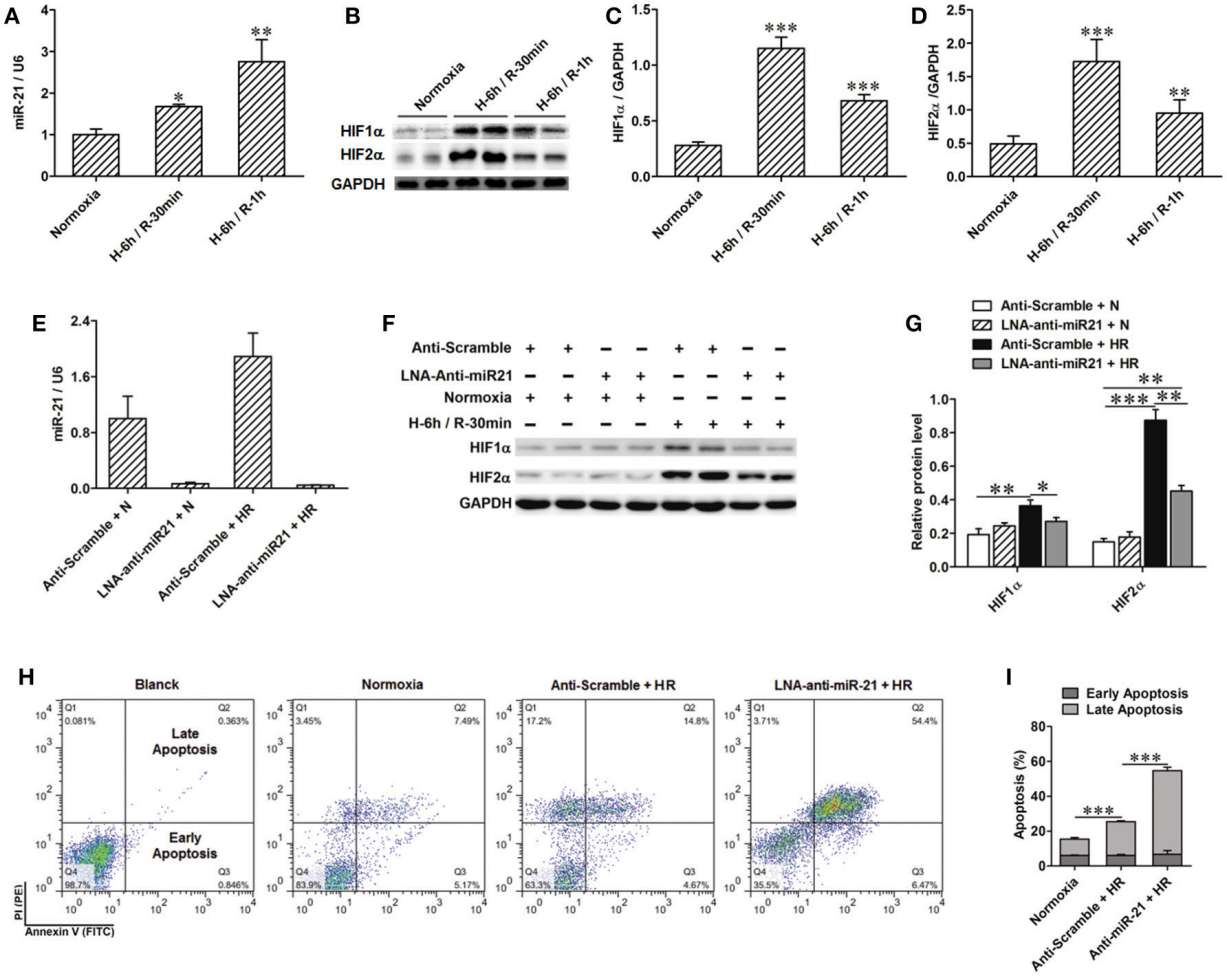
Anti-miR21 attenuated HR induced up-regulation of HIF-1α and HIF-2α and aggravated HR induced apoptosis of HK-2. Expression of miR-21, HIF-1α and HIF-2α in the HK-2 cells under hypoxia (6 h)/ reoxygenation (30 min or 1 h) condition were detected. **(A)** expression level of miR-21 in the HK-2 cells was increased from 30 min after reoxygenation compared with normoxia condition. **(B)** Protein level of HIF-1α and HIF-2α were also elevated in the HR-treated HK-2 cells. **(C,D)** group data from **F**, *n* = 6, *p < 0.05, **p < 0.01, ***p < 0.01 compared with normoxia condition. **(E)** Transfection of 50 nM LNA-modified anti-miR-21 to HK-2 cells significantly inhibited expression of miR-21 even under normoxia or HR condition. **(F)** Inhibition of miR-21 attenuated HR induced up-regulation of HIF-1α and HIF-2α. **(G)** Group data from F. *p < 0.05, **p < 0.01, ***p < 0.01, *n* = 6. **(H)** Apoptosis of the HK-2 cells was accessed by annexin-V-FITC/PI staining and determined by flow cytometry. Knockdown of miR-21 attenuated HR induced apoptosis of HK-2 cells. **(I)** Group data from **(H)**. *** < 0.001, *n* = 3.

Renal epithelial cells are specifically sensitive to hypoxia. We analyzed the expression of miR-21 in HK-2 cells treated by HR. HR increased miR-21, HIF-1a, and HIF-2α expression in HK-2 cells (Figures 4A–D). To investigate the effect of miR-21 on HIF-1α and HIF-2α, miR-21 was inhibited by administration of LNA-anti-miR-21. The inhibition of miR-21 expression was confirmed by PCR. Knockdown of miR-21 reduced the expression of both HIF-1α and HIF-2α (Figures 4E–G). Additionally, HR increased late apoptosis of HK-2 cells, and miR-21 inhibition increased the effect of HR on apoptosis (Figures 4H,I).

**Figure 5:**
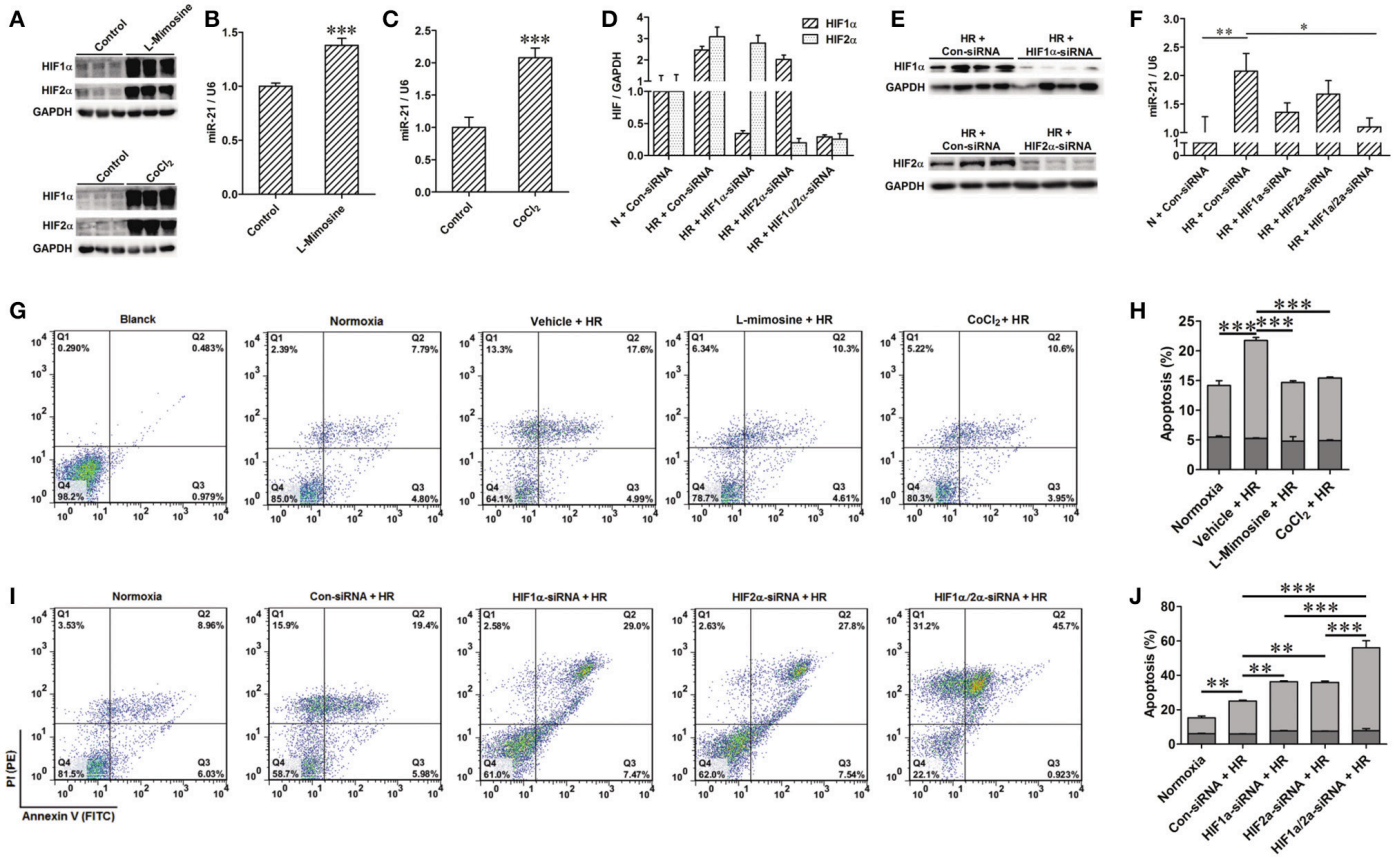
Activation of HIF protected HK-2 injury by mediating miR-21. **(A–C)** Hydrolysis of HIF was inhibited by 500 μM L-Mimosine or 150 μM CoCl_2_. Elevated expression of HIF increased expression of miR-21. **(D,E)** Interfered expression of HIF1α and/or 2α by transfected with HIF1α and/or 2α siRNA to HK-2 cells under hypoxia/reoxygenation and normoxic conditions was confirmed by qRT-PCR and western blotting. **(F)** Interference expression of HIF1α and/or 2α modulated elevated expression of miR-21 induced by HR. **(G)** Apoptosis of the HK-2 cells was accessed by annexin-V-FITC/PI staining and determined by flow cytometry. Treatment of HK-2 cells with L-Mimosine or CoCl_2_ attenuated HR induced apoptosis. **(H)** Group data from **(E)**. **(I)** HR increased late apoptosis of HK-2 cells, after inhibiting HIF1α and/or 2α, the effect of HR on apoptosis was enhanced. **(J)** Group data from I. *P < 0.05, **P < 0.01, *** < 0.001, *n* = 3 for flow cytometry, *n* = 6 for others.

To confirm the positive regulation of miR-21 by HIF, the PHD inhibitors L-Mimosine and CoCl_2_ were used to inhibit the degradation of HIF in HK-2 cells (Figure 5A). The expression of miR-21 was increased (Figures 5B,C). HIF-1α and HIF-2α expression was inhibited by transfection of HIF-1α and/or HIF-2α siRNAs (Figures 5D,E). Silencing of HIF-1α or HIF-2α individually or simultaneously suppressed the increase in miR-21 evoked by HR. However, the effect of simultaneously blocking both HIFs yielded the most striking results (Figure 5F). Hypoxia stimulated miR-21 expression through both HIF-1α and HIF-2α. HK-2 cell apoptosis induced by HR was attenuated by L-mimosine and CoCl_2_ (Figures 5G,H). HR increased late apoptosis of HK-2 cells, and inhibition of HIF1α and/or 2α increased the effect of HR on apoptosis. More interestingly, the apoptogenic effect of silencing of HIFs simultaneously was greater than inhibiting them individually, indicating that both HIF-1α and HIF-2α are involved in the protection of HK-2 cells from apoptosis (Figures 5I,J).

### MiR-21 regulates activation of PTEN/AKT/mTOR/HIF and PDCD4/NF-κb pathways in renal epithelial cells

**Figure 6:**
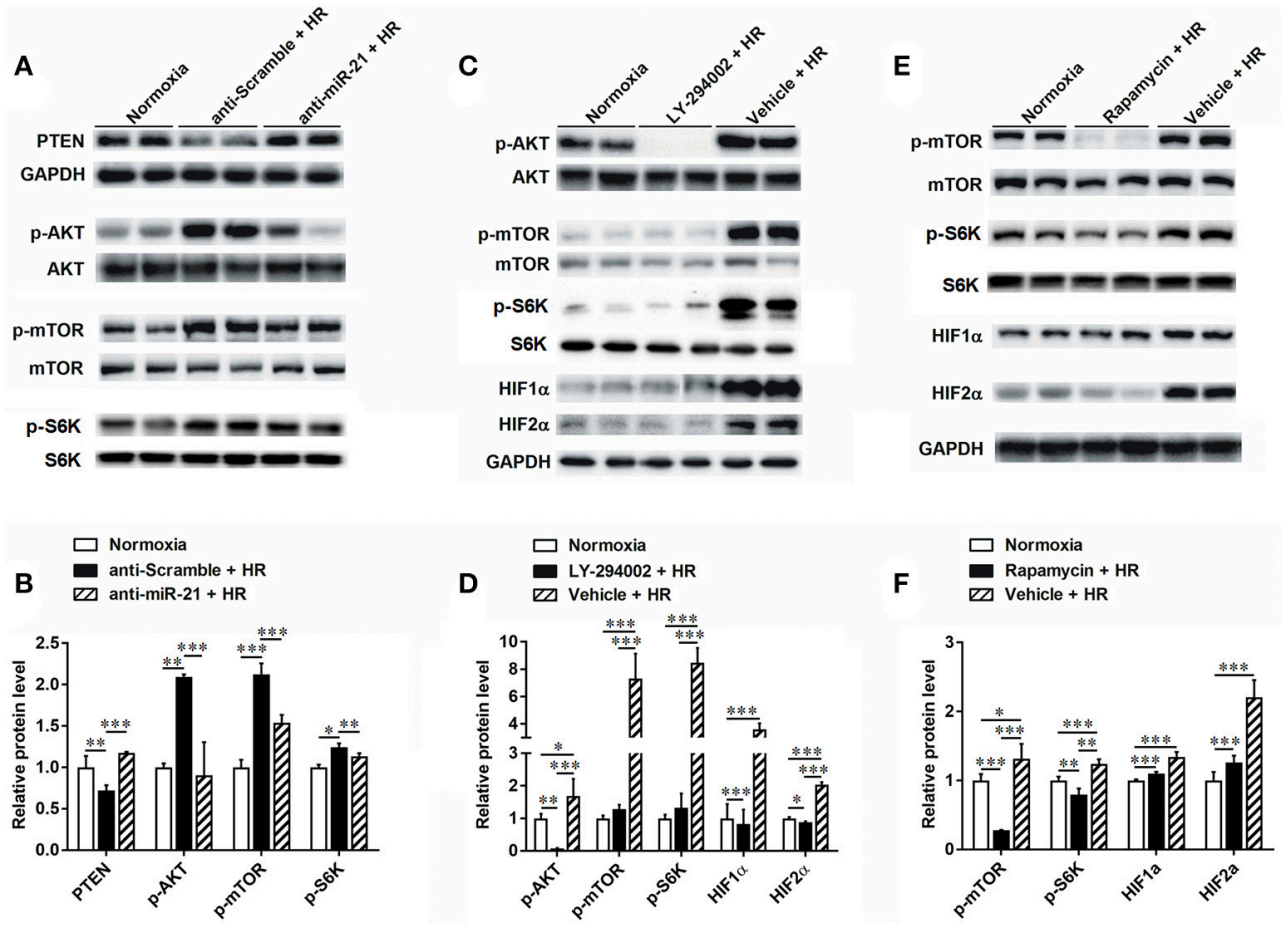
HR inducible miR-21 mediated HIF-1α and HIF-2α expression through PTEN/AKT/mTOR pathway. **(A)** HK-2 cells were transfected with LNA-modified anti-miR-21 or the scrambled control under hypoxia/reoxygenation and normoxic conditions. Western blotting was performed to analyze the expression of PTEN and phosphorylation of AKT and mTOR. HR treatment inhibited expression of PTEN, otherwise increased phosphorylation of both AKT and Mtor. **(B)** Group data from **(A)**. **(C)** Phosphorylation of AKT was blocked by treating HK-2 cells with 50 μM LY294002 during HR. Inhibition of AKT prevents phosphorylation of mTOR and inductions of HIF-1α and HIF-2α under HR condition. **(D)** Group data from **(C)**. **(E)** Phosphorylation of mTOR was blocked by treating HK-2 cells with 10 nM rapamycin during HR. Inhibition of mTOR reduced HIF-1α and HIF-2α stabilization. **(F)** Group data from **(E)**. *p < 0.05, **p < 0.01, ***p < 0.01, *n* = 6.

To clarify the molecular mechanism involved in the renal protective effect of miR-21, we assessed downstream targets of mir-21. PTEN is a popular target of miR-21. We found that expression of PTEN was decreased under conditions of hypoxia. However, inhibition of miR-21 resulted in an increase in PTEN (Figures 6A,B). Our results indicated that phospho-Akt and phospho-mTOR were increased under conditions of hypoxia and reduced upon miR-21 inhibition (Figures 6A,B). Moreover, the use of LY294002 efficiently reduced phospho-mTOR, HIF-1α, and HIF-2α expression (Figures 6C,D). Additionally, Inhibition of mTOR via rapamycin treatment prevents HIF-1α and HIF-2α stabilization under hypoxia (Figures 6E,F). These results demonstrate that miR-21 up regulated HIF-1α and HIF-2α expression via the regulation of PTEN/Akt/mTOR signaling pathway activation.

**Figure 7:**
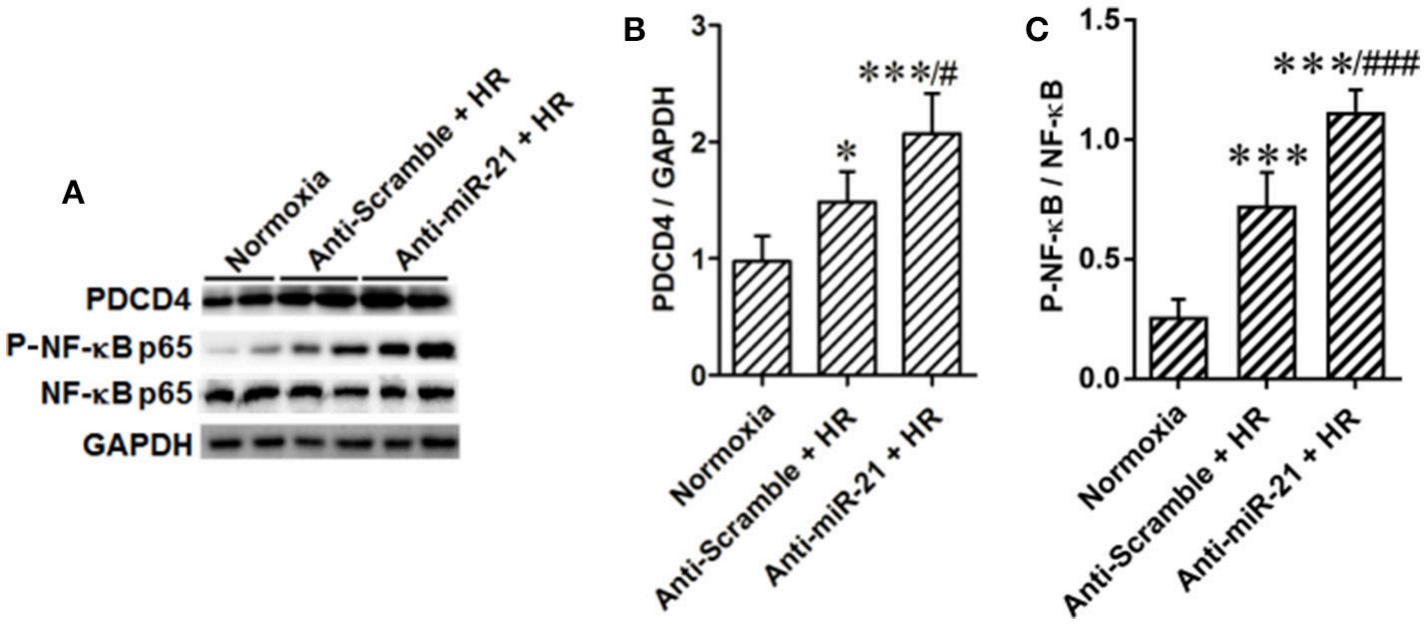
Knockdown of miR-21 activates PDCD4/NF-κB pathway *in vitro*. The effect of miR-21 on activation of PDCD4/NF-κB pathway was assessed in HR cultured HK-2 cells. **(A–C)** Expressions of PDCD4 and NF-κB were increased in the HK-2 cells challenged by HR and knockdown of miR-21 aggravated this effect. *P < 0.05, *** < 0.001, compared with Normoxia; ^#^*P* < 0.051, ^###^*P* < 0.001, compared with Anti-scramble + HR, *n* = 6.

In addition, we found that the PDCD4/NF-κB pathway is activated in the kidney after IR. To specify cell type functions of NF-κB, we measured activation of PDCD4/NF-κB pathways in tubular epithelia cells. HR increased PDCD4 and NF-κB levels in HK-2 cells; anti-miR21 further increased these levels (Figures 7A–C).

**Figure 8:**
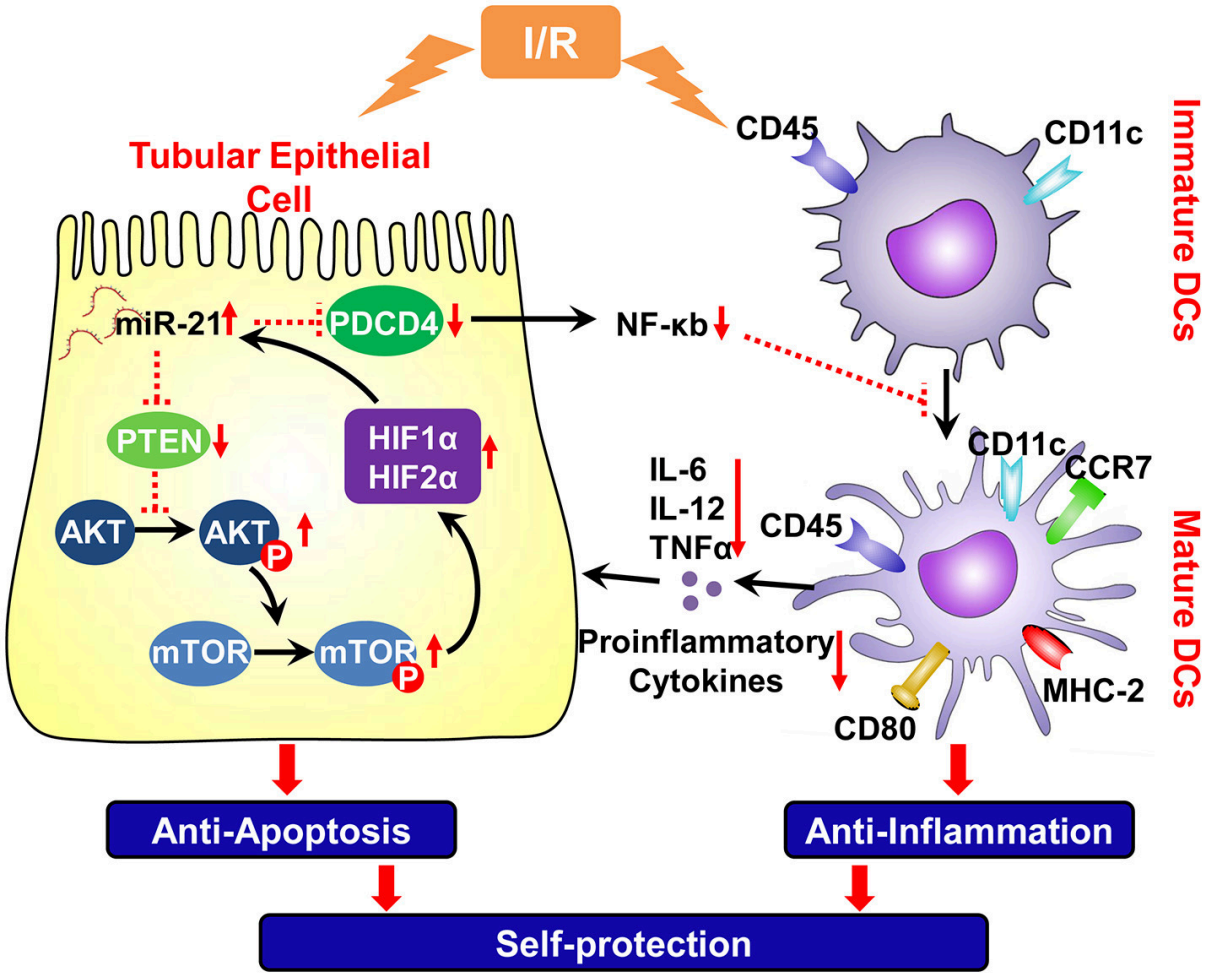
Schema of IR induced miR-21 on the renal self-protection and the involved underlying mechanism. Ischemia/reperfusion elevated expression of miR-21 in the tubular epithelial cells, which prevent kidney injury by resisting apoptosis of epithelial cells directly and by inhibiting inflammatory reaction evoked by mature DCs. A positive feedback loop between miR-21 and HIF-1α/2α was mediated by PTEN/Akt/mTOR pathway, which is involved in the reduction of epithelial apoptosis induced by ischemia/reperfusion. Upon injury of epithelium, ischemia/reperfusion also triggers inflammation by inducing maturation of DCs. Up-regulation of miR-21 also down-regulates the PDCD4 and then NF-κB that is an important factor to promote the maturation of dendritic cells. Thus, IR induced miR-21 contributes to anti-inflammatory reaction and renoprotection by inhibiting maturation of DCs. PTEN, tensin homolog deleted on chromosome 10; PDCD4, proapoptotic target effectors programmed cell death protein 4; Akt, protein kinase B; p-Akt, phospho-Akt; mTOR, mammalian target of rapamycin; p-mTOR, phospho- mTOR; HIF, hypoxia-inducible factor; CCR-7, chechemokinereceptor7; NF-κB, nuclear factor-k-gene binding k; TNF-α, Tumor Necrosis Factor-alpha. Red vertical arrows: “↓” means decrease, “↑” means increase. The red dotted line means inhibiting effect.

These results indicated that ischemia/reperfusion increased miR-21 expression in tubular epithelial cells, which prevent kidney injury via the prevention of epithelial cell apoptosis through the PTEN/AKT/mTOR/HIF pathway directly and by inhibiting the inflammatory reaction evoked by mature DCs through the PDCD4/NF-κB pathway (Figure 8).

## Discussion

The kidney is especially prone to ischemia. The initial HR injury of renal parenchyma and subsequent activation of immune responses results in renal damage. Our previous study indicated that miR-21 mediates the protective effects of xenon or delayed ischemia preconditioning for IR and sepsis-induced kidney injury (Xu et al., 2012; Jia et al., 2013, 2015; Jiao et al., 2016). However, the effect of miR-21 on renal IRI remains unclear. Our results indicated that miR-21 is critical for epithelial cell survival under ischemic conditions. The data suggest that miR-21 expression was significantly up-regulated by ischemia *in vivo* or by hypoxia *in vitro*. A feedback loop exists between miR-21 with both HIF1α and HIF2α. Inhibition of this feedback loop exacerbates IR-induced tubular epithelia cell apoptosis. DCs are a major component of immunocytes in kidney, which play a critical role in initiating an early immune response against various noxious stimulation and inducing immunological tolerance (Li et al., 2012). We demonstrated that miR-21 knockdown exacerbated IR-induced maturation of DCs and increased the secretion of pro-inflammatory cytokines, such as IL-12, IL-6, and TNF-α. Thus, the present study provided definitive evidence that the protective role of miR-21 in the kidney IRI was mediated by inhibiting apoptosis of tubular epithelial cells and reducing DC maturation.

MiR-21 was up-regulated in several distinct animal models of kidney diseases as well as both human AKI and chronic kidney disease (CKD) (Loboda et al., 2016). Thus, miR-21 is considered a potential biomarker for cardiac surgery-associated acute kidney injury, hypertensive kidney injury and fibrosis (Gaede et al., 2016; Arvin et al., 2017; Chen et al., 2017). However, the effect of miR-21 on renal injury remains controversial. We previously demonstrated that ischemic and xenon preconditioning induced up-regulation of miR-21, which mediated renal protective effects by inhibiting inflammation and cell apoptosis and promoting angiogenesis (Jia et al., 2015; Xu et al., 2017a,b). Similarly, up-regulation of miR-21 by ghrelin can ameliorate IR-induced AKI (Zhang and Shu, 2016). In the present study, we also found that expression of miR-21 was increased by ischemia *in vivo* and *in vitro*. In addition, blocking miR-21 exacerbated IR-induced AKI. The up-regulation of miR-21 represents a self-protective mechanism against ischemic injury. In contrast, up-regulation of miR-21 is associated with renal fibrosis in several mouse models, including UUO and diabetic kidney disease (Zarjou et al., 2011; Loboda et al., 2016; Sun et al., 2018). In patients with IgA nephropathy, miR-21 expression was significantly increased during the development of fibrotic lesions and is associated with renal survival (Hennino et al., 2016). Correspondently, miR-21 was regarded as a potential target to treat renal fibrosis. The role of miR-21 in renal injury is complex. Temporary elevation of miR-21 may be protective for kidneys to promote the renal repair; however, long-term miR-21 overexpression may be detrimental by inducing renal fibrogenesis following IR injury.

The need for effective AKI therapy is urgent given the marked increase in the incidence and unacceptably high morbidity. Numerous factors contribute to the development of AKI. Among these factors, IRI is the most common. HIFα induction, including HIF1α and HIF2α, during renal ischemia promotes tissue adaptation and survival (Hill et al., 2008). Our current study revealed that HIF1α and HIF2α protein levels were detected in HK-2 cells, which is consistent with findings in previous studies (Schönenberger et al., 2016). Our previous studies demonstrate that the interaction between miR-21 and HIF1α is involved in the renoprotective mechanism of delayed ischemic and xenon preconditioning. The present study demonstrates that miR-21 and both HIFs (1α and 2α) are inducible following IRI. MiR-21 affected the expression of HIFs as demonstrated by the observation that anti-miR-21 attenuated HIF overexpression induced by IR.

Between the two HIFs, the effect of HIF1α on renal ischemia has been extensively studied. The interaction between miR-21 and HIF1α in response to hypoxia in cardiomyocytes was previously proposed (Liu et al., 2014). The Akt/mTOR signaling pathway was activated in response to hypoxia, which leads to accumulation of HIF1α and mediates proximal tubule cell survival (Conde et al., 2012). PTEN, an inhibitor of the Akt/mTOR pathway, is a target gene of miR-21 (Liu et al., 2011). In the current study, we demonstrated that PTEN was reduced and that the Akt/mTOR pathway is activated following IRI. Inhibition of miR-21 naturalized the effect of IR on the PTEN/Akt/mTOR pathway. We also observed that blocking the Akt/mTOR pathway using LY 294002 and rapamycin prevented HIF induction during HR in HK-2 cells. Our results indicated that miR-21 modulated the expression of HIFs by targeting the PTEN/Akt signaling pathway, which is involved in the response of the kidney to IRI. In addition, subsequent knockdown of HIF1α and/or 2α reduced induction of miR-21 under HR conditions. Moreover, activation of HIFs by CoCl_2_ and L-mimosine increased miR-21 expression. The effect of knocking down both HIFs on the expression of miR-21 greater than the inhibitory effect produced by either agent alone. This result implied that miR-21 was regulated by both HIFs. A HIF1α binding site was previously identified in the miR-21 promoter (Liu et al., 2014). However, the HIF2α binding site in the miR-21 promoter was not predicted to date. Additional efforts are required to elucidate how HIF2α regulates miR-21 expression.

Beyond direct cell damage, IR triggers the innate immune response in the kidney and contributes indirectly to tubular cell necrosis and renal failure. Kidney resident DCs, the predominant leukocyte subset in the kidney, reside in the interstitium in close proximity to tubular epithelial cells and respond to changes in the local microenvironment (Soos et al., 2006; Li et al., 2012). Depletion of DCs protects kidneys from IRI, whereas the injection of WT DCs exacerbates IRI (Li and Okusa, 2010; Bajwa et al., 2012). Mature DCs contribute to renal inflammation and exacerbate IRI; however, immature DCs lead to immune tolerance (Li et al., 2012). CD45^+^/*CD*11*c*^+^ cells are resident renal DCs undergoing maturation in the kidney following IRI (Dong et al., 2007). MHC-2, CD80 and CCR7 expression is up-regulated when DCs are activated (Morelli and Thomson, 2007; Hochheiser et al., 2013; Yang et al., 2015). As confirmed in the present study, the percentage of CD45^+^/*CD*11*c*^+^ cells with mature markers CD80 and MHC-2 and CCR7 expression was increased after IRI. It has been reported that miR-21 inhibited maturation of DCs by negatively regulating CCR7 expression (Al Akoum et al., 2015). Although some researcher believe that miR-21 is nonfunctional with activity maintained below a threshold required for binding to its target mRNAs under normal conditions (Chau et al., 2012; Loboda et al., 2016), the current study indicates that endogenous miR-21 maintains an immature state of kidney resident DCs given that anti-miR-21 treatment of sham operation mice resulted in improved DC maturation. Another study also mentioned that the abundance of miR-21 is negatively correlated with DC maturation (Al Akoum et al., 2015). In addition, miR-21 inhibition exacerbated IR-induced DC maturation and kidney injury. These results implied that the up-regulation of miR-21 by IR relieved kidney injury. Thus, DCs offer an important therapeutic target to minimize tissue injury.

A broad range of proinflammatory mediators is elucidated within the injured kidney and associated with the severity of renal structural and functional disturbance following IRI. Previous experiments indicated that mature DCs secreted substantially vast amounts of the cytokines IL-12, IL-6, and TNF-α (Kapsenberg, 2003; Dong et al., 2007). IL-12, IL-6, and TNF-α play a nonredundant role in determining the severity of acute kidney injury following IRI (de Paiva et al., 2009; Chen et al., 2011; Susantitaphong et al., 2013). The current results also indicate that IL-12, IL-6, and TNF-α production is increased in IRI kidney. Furthermore, we observed that anti-miR-21 further increased production of IL-12, IL-6, and TNF-α in kidneys subjected to IR challenge. Taken together, we favor the conclusions that deterioration of renal IRI caused by anti-miR-21 may contribute to the increased levels of pro-inflammatory cytokine secretion from mature DCs. However, additional investigations are needed to clarify the effect of miR-21 on renal resident DCs.

It was reported that a miR-21 precursor blocked NF-κB activity by targeting PDCD4 in response to lipopolysaccharide (LPS) (Sheedy et al., 2010). MiR-21 mimics attenuated TNF-α-induced NF-κB activity in renal inner medullary collecting duct cells (Lin et al., 2017). Sustained activation of NF-κB enhances longevity and promotes DC maturation (Jimenez et al., 2010). However, inhibition of NF-κB blocks DC maturation and protects against renal IRI (Clement et al., 2014; Lobo et al., 2015). Our previous study also indicates that xenon preconditioning inhibited PDCD4 expression and NF-κB activity to protect LPS-induced kidney injury by increasing miR-21 levels (Jia et al., 2015). Our evidence revealed that IR increased PDCD4 and NF-κB expression and DC maturation; moreover, inhibiting miR-21 increased the effects of IR and aggravated the kidney injury. We conclude that miR-21 and its signaling pathways PDCD4/NF-κB are involved in IR-induced kidney injury by mediating DC maturation.

Previous findings together with our data suggest that IR-inducible miR-21 expression prevents epithelial cells via a feedback interaction with HIF (miR-21/PTEN/AKT/mTOR/HIF/miR-21) and by inhibiting DC maturation via the PDCD4/NF-κB pathway. Up-regulation of miR-21 under IR conditions is involved in a self-protective mechanism of the kidney.

## Author contributions

NS and TZ conceived and carried out experiments, analyzed data, searched literature and generated figures. ZL, XX, and JH carried out experiments and collected data. NS, XY, PJ, YF, and JT interpreted data. XD and JT designed study, interpreted data and wrote the manuscript.

### Conflict of interest statement

The authors declare that the research was conducted in the absence of any commercial or financial relationships that could be construed as a potential conflict of interest.

## Abbreviations

PTEN: phosphatase and tensin homolog deleted on chromosome ten
AKT: protein kinase B
mTOR: mammalian target of rapamycin
HIF: hypoxia-inducible factors
PDCD4: programmed cell death 4
NFκ-B: nuclear factor-kappa B
IL: interleukin
TNF-α: tumor Necrosis Factor-alpha
DCs: dendritic cells
IR: ischemia/reperfusion
HR: hypoxia/reoxygenation
AKI: acute kidney injury
IRI: ischemia/reperfusion injury
HK-2 cells: human proximal tubular cell line
miR: MicroRNA.
